# The Physician’s Visiting List (Lindsay & Blakiston’s) for 1892

**Published:** 1891-12

**Authors:** 


THE PHYSICIAN'S VISITING LIST (LINDSAY & BLAKISTON'S) FOR 1892. The forty-first year of its publication. Philadelphia: P. Blakiston, Sons & Co., 1012 Walnut street. Price, from $1.00 to $3.00, according to size.

This standard Visiting List is one of the first to put in its appearance, and is easily seen to be one of the best. It is simple in its arrangement, and at the same time sufficiently large to meet all the requirements of the busy physician. It is published in several fashions, to-wit: A monthly edition, a perpetual edition, an interleaf edition, and a regular edition. It is bound in substantial morocco, and each book is supplied with one of Blakiston's lead pencils with eraser. We need not enumerate the table of contents, for it does not differ greatly from others in respect to this preliminary matter, but for compactness, simplicity of arrangement, and completeness of detail, it is not excelled by any visiting list in the market.

